# Possible progressive multifocal leukoencephalopathy and active multiple sclerosis under dimethyl fumarate: the central role of MRI in informing therapeutic decisions

**DOI:** 10.1186/s12883-021-02165-0

**Published:** 2021-04-05

**Authors:** Elena Augusta Vola, Maria Petracca, Sirio Cocozza, Marcello De Angelis, Antonio Carotenuto, Giuseppe Pontillo, Vincenzo Brescia Morra, Enrico Tedeschi, Roberta Lanzillo

**Affiliations:** 1 Department of Advanced Biomedical Sciences, “Federico II” University, Naples, Italy; 2 Department of Neurosciences, Reproductive and Odontostomatological Sciences, “Federico II” University, Naples, Italy

**Keywords:** Progressive multifocal leukoencephalopathy, Dimethyl fumarate, Neuroimaging, MRI, Risk stratification, Diagnostic criteria, Case report

## Abstract

**Background:**

Progressive multifocal leukoencephalopathy (PML) can rarely occur in Multiple Sclerosis (MS) patients undergoing dimethyl fumarate (DMF) treatment. Our case stresses the limits of current diagnostic and stratification risk criteria, highlighting the potential role of Magnetic Resonance Imaging (MRI) in advising clinical choices.

**Case presentation:**

A 54 years old MS male patient treated with DMF, after 3 years of clinical stability developed a subacute clinical worsening. He had no severe lymphopenia but MRI signs suggestive of a coexistence of PML and MS activity. Although his viral title was negative, DMF was discontinued, with clinical and radiological improvement.

**Conclusions:**

This case highlights the challenges behind PML diagnosis, especially in patients not fulfilling the risk stratification criteria and that might present with concurrent disease activity, stressing the potential role of MRI in informing therapeutic decisions.

## Background

Progressive multifocal leukoencephalopathy (PML), caused by the John Cunningham polyomavirus (JCV), represents a rare but known safety risk under dimethyl fumarate (DMF) treatment [1]. So far, a total of 11 PML cases have been reported in MS patients under DMF treatment. All but three patients developed PML associated with prolonged severe lymphopenia [1–3]. Brain Magnetic Resonance Imaging (MRI) is a sensitive diagnostic tool for the PML detection [4], usually showing a heterogeneous pattern characterized by multifocal, often confluent, subcortical lesions with frequent involvement of the U-fibers [5]. Nevertheless the differential diagnosis between MS activity and PML can prove challenging, as highlighted by the proposal of early imaging biomarkers based on Susceptibility Weighted Imaging and Quantitative Susceptibility Mapping [6] and of applying high-resolution imaging to differentiate early PML- from MS-induced [7]. Here, we describe a case of clinical worsening in a MS patient under DMF therapy, in course of moderate lymphopenia, with negative viral title but with MRI signs highly suggestive of PML and concomitant MS disease activity.

## Case presentation

A 54 years old Relapsing-Remitting MS male patient presented to the Neurology Department in July 2019 for subacute clinical worsening. He had been diagnosed in September 2016,and treated with DMF since then. Along with DMF, he had started mirtazapine treatment for concurring depression. From the disease onset, the patient had no signs of disease activity, presenting minor stable clinical findings at the neurological examination (Expanded Disability Status Scale -EDSS- score = 2.5), characterized by slight hands telekinetic tremor and mild lower limbs hypoesthesia. His absolute lymphocyte counts (ALC) under treatment always ranged between 623 and 678 cells/μL.

His neurologic examination in July 2019 showed cerebellar ataxia, dysarthria and lower limbs weakness (EDSS = 3.5). Brain MR showed imaging features highly suggestive for PML overlapping with an enhancing MS lesion. In particular, compared to the previous MR exam, several new hyperintense and enlarging lesions were found on Fluid Attenuated Inversion Recovery (FLAIR) images, in absence of restricted diffusion. Among these, 4 confluent hyperintense lesions, with involvement of the U-fibers,were present, with 3 showing a punctate peripheral gadolinium enhancement along with a hypointense subcortical rim at the Spoiled Gradient Recalled (SPGR) sequence, while 1 lesion showed a well-defined area of contrast enhancement without SPGR hypointensity.

**Fig. 1:**
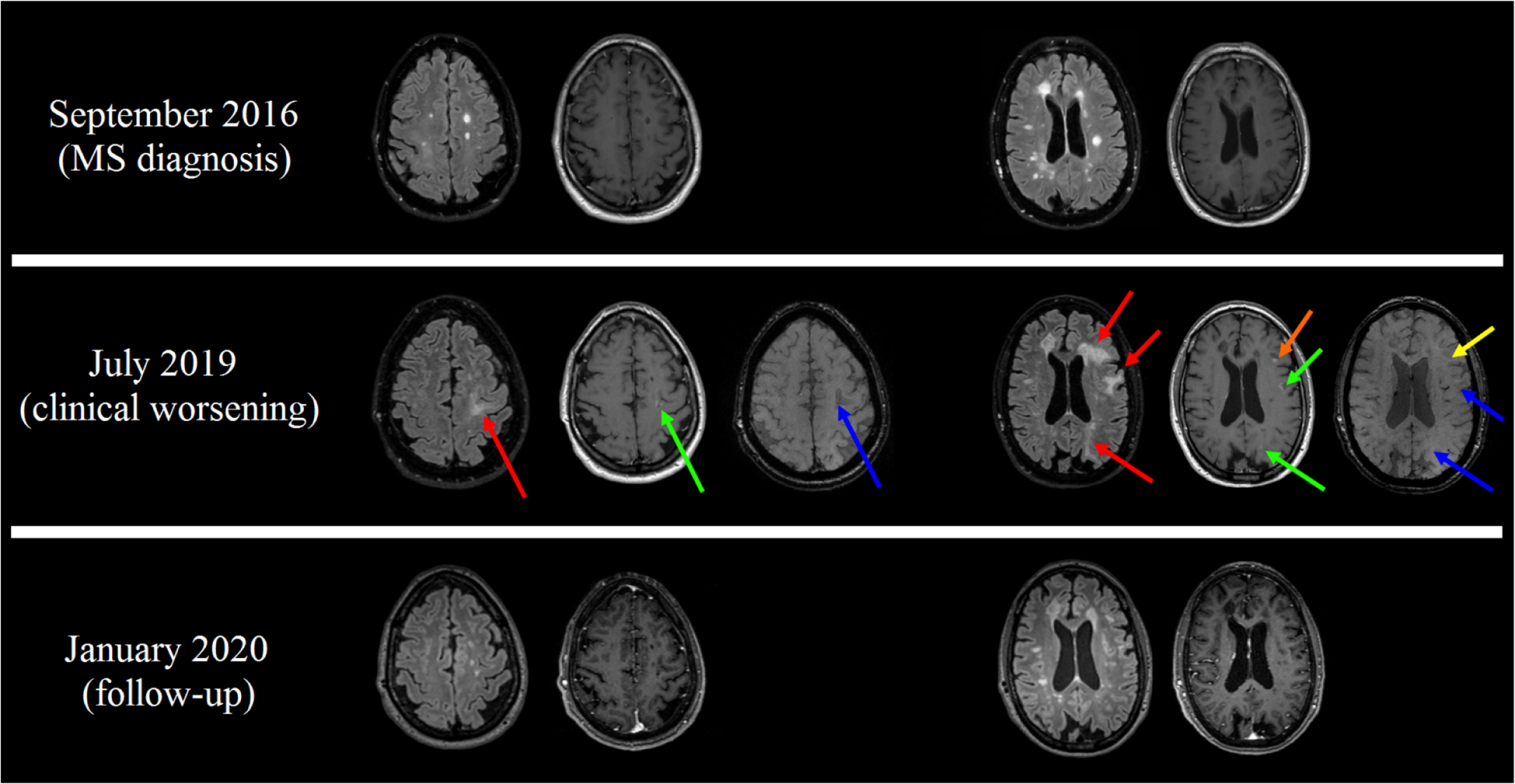
Selected MRI findings in our Multiple Sclerosis (MS) case. At the baseline exam (September 2016, upper row) a typical radiological MS pattern was present. When the patient presented a subacute clinical worsening (July 2019, middle row), brain MRI scan showed the presence of four new confluent Fluid Attenuated Inversion Recovery (FLAIR) hyperintense lesions (red arrows), with involvement of the U-fibers, a sharp border between the superficial aspect of the lesion and the overlying cortex and an ill-defined deeper border. Three of these lesions showed a punctate peripheral contrast-enhancement (green arrows) and a mild, but consistent, hypointense subcortical rim in Spoiled Gradient Recalled (SPGR) images (blue arrows), suggestive of PML lesions. Along with these lesions, a left frontal oval-shaped lesion showed a different pattern of contrast enhancement compared to the other lesions (orange arrow), in absence of hypointense signal in SPGR images (yellow arrow), more suggestive of active MS rather than a PML lesion. At a follow-up examination (January 2020, lower row), the PML lesions showed an almost complete resolution, in absence of significant sequelae, while the oval-shaped FLAIR hyperintense lesion was still present, although reduced in volume

Representative imaging findings are shown in Fig. 1. Written informed consent was obtained from the patient for the publication of this report.

ALC on admission was 620 cells/μL, while cerebrospinal fluid (CSF) proteins and cells were unremarkable. JCV-PCR (Polymerase Chain Reaction) in two CSF samples, obtained from the same spinal tap but analyzed in different laboratories, resulted negative. Extensive microbiological tests, including HIV, were unremarkable. Given the mild clinical course, no brain biopsy was performed. DMF therapy was discontinued, while mirtazapine administration was carried on.

In January 2020 a brain MR demonstrated stable MS lesion load, with resolution of PML signs and no evidence of immune reconstitution inflammatory syndrome. ALC ranged between 790 and 950 cells/μL after treatment discontinuation. The patient showed a clinical resolution of gait ataxia and dysarthria, with a stability of the hand telekinetic tremor and mild lower limbs hypoesthesia. At this point, interferon-beta-1a treatment was started, with clinical and radiological stability.

## Discussion and conclusions

The concomitant occurrence of PML and disease activity in MS is a rare but possible event, and, in this case, prompt recognition is necessary to inform therapeutic decisions. Here we report the case of a “possible PML” overlapping with MRI MS activity in a patient under DMF without severe lymphopenia. Although in 2020 the European Medicines Agency recommended intensification of controls in patients with sustained lymphopenia, especially when concomitant risk factors for PML are present (therapy duration, decrease in CD4+ and CD8+ T cell counts), in 2019 PML risk stratification under DMF treatment was still based on age and lymphopenia levels, with moderate lymphopenia (severe:< 500 cells/μL, moderate:500-800cells/μL, mild: 800-900cells/μL) prompting a re-evaluation of the patients’ risk-benefit balance. In our case the risk of PML and the possibility to switch to another first-line therapy were discussed with the patient. Given the concomitant presence of hypertension, teriflunomide was not considered and the patient refused injectable drugs, which led to DMF continuation. When subacute neurological worsening occurred, in presence of moderate lymphopenia with brain MRI suggestive of PML, a spinal tap was performed.

Routinely, PML diagnosis is based on the demonstration of JCV-DNA in the CSF, using PCR, in presence of suggestive features [4]. Although JCV-PCR is highly specific (92–99%), false negatives can occur, given the relative low and wide sensitivity of the technique (74–93%), possibly due to the low CSF titers of JCV-DNA [8].When one among the virologic, radiologic or clinical criteria is missing, the diagnosis of “possible PML” may be defined [4].

In our case, although the PCR showed negative results, neuroimaging findings were highly suggestive of PML. Indeed, MRI showed 3 typical multifocal confluent subcortical FLAIR hyperintense lesions, different from the usual relapsing MS lesions. A subcortical U-fibers involvement was evident, with the gradient sequence showing a hypointense rim of the U-fibers adjacent to the white matter lesions. These features, coupled to a punctuate marginal contrast enhancement, are considered highly suggestive of PML^6^. Another new lesion presented completely different features, typical for an active MS lesion: ovoid shape, with well-defined contrast enhancement and margins. In our patient, despite the negativity of the spinal tap, in particular the identification of the highly specific punctuate pattern at MRI [9] contributed to the diagnosis of possible PML and guided our therapeutic choices, resulting in a favorable clinical outcome.

Given the relatively scarcity of reports about the development of DMF-associated PML in MS patients, no treatment or management guidelines are yet available. Nevertheless, growing importance has been recently given to MRI signs of PML in MS patients not only in the diagnosis, but also in the management of these patients. Indeed, a recent study suggested that the possibility to withdraw the treatment in case of radiologically suspected PML, even in those cases without a confirmation at the CSF analysis, and to perform a close imaging and clinical monitoring before starting a new MS therapy [10].

Emerging studies reports clinical improvement attributable to an off-label use of mirtazapine in the treatment of PML, in HIV positive patients, autoimmune patients under immunosuppressive treatment and in natalizumab-associated PML [11, 12]. In-vitro studies have demonstrated that JCV infects oligodendrocytes via serotonin 5HT_2A_-receptors, suggesting that 5HT_2−_ antagonists as mirtazapine might improve PML course [13]. In this regard, we anecdotally report that mirtazapine was not discontinued in our patient, which showed a mild PML course, probably also in consideration of other factors such as the moderate lymphopenia and the lack of previous immunosuppressive treatment.

In conclusion, our case highlights how PML diagnosis remains challenging, especially in patients that do not fully meet the risk stratification or diagnostic criteria and that might present with concurrent disease activity. Although JCV demonstration is the main diagnostic criterion, JCV-PCR has proved to be not completely reliable and negative results cannot exclude PML diagnosis. In these cases, neuroimaging plays a central role in the diagnostic process and the presence of imaging features highly suggestive for PML should be thoroughly evaluated. In our case, an accurate evaluation of brain MRI scans was the key to the discrimination of MR MS activity from PML lesions, which in turn was crucial for treatment decisions. Imaging findings drove DMF discontinuation, notwithstanding the negative JCV-PCR, with clinical benefits.

## Data Availability

Data sharing is not applicable to this article as no datasets were generated or analyzed during the current study.

## Abbreviations

PML: Progressive Multifocal Leukoencephalopathy
DMF: Dimethyl fumarate
MRI: Magnetic Resonance Imaging
JCV: John Cunningham polyomavirus
EDSS: Expanded Disability Status Scale
ALC: Absolute lymphocyte counts
FLAIR: Fluid Attenuated Inversion Recovery
SPGR: Spoiled Gradient Recalled
CSF: Cerebrospinal fluid
PCR: Polymerase Chain Reaction
